# Efficacy of Adjuvant 5-Fluorouracil Therapy for Patients with EMAST-Positive Stage II/III Colorectal Cancer

**DOI:** 10.1371/journal.pone.0127591

**Published:** 2015-05-21

**Authors:** Yasushi Hamaya, Carla Guarinos, Stephanie S. Tseng-Rogenski, Moriya Iwaizumi, Ritabrata Das, Rodrigo Jover, Antoni Castells, Xavier Llor, Montserrat Andreu, John M. Carethers

**Affiliations:** 1 Division of Gastroenterology, Department of Internal Medicine, University of Michigan, Ann Arbor, Michigan, United States of America; 2 Unidad de Gastroenterologia, Hospital General Universitario de Alicante, Alicante, Spain; 3 Biostatistics Department, School of Public Health, University of Michigan, Ann Arbor, Michigan, United States of America; 4 Department of Gastroenterology, Hospital Clínic, Centro de Investigación Biomédica en Red en Enfermedades Hepáticas y Digestivas (CIBERehd), IDIBAPS, University of Barcelona, Barcelona, Spain; 5 Division of Gastroenterology, Department of Medicine, Yale University, New Haven, Connecticut, United States of America; 6 Department of Gastroenterology, Hospital del Mar, IMIM (Institut Hospital del Mar d'Investigacions Mèdiques), Pompeu Fabra University, Barcelona, Catalonia, Spain; Shanghai Jiao Tong University School of Medicine, CHINA

## Abstract

Elevated Microsatellite Alterations at Selected Tetranucleotide repeats (EMAST) is a genetic signature found in up to 60% of colorectal cancers (CRCs) that is caused by somatic dysfunction of the DNA mismatch repair (MMR) protein hMSH3. We have previously shown *in vitro* that recognition of 5-fluorouracil (5-FU) within DNA and subsequent cytotoxicity was most effective when both hMutSα (hMSH2-hMSH6 heterodimer) and hMutSβ (hMSH2-hMSH3 heterodimer) MMR complexes were present, compared to hMutSα > hMutSβ alone. We tested if patients with EMAST CRCs (hMutSβ defective) had diminished response to adjuvant 5-FU chemotherapy, paralleling *in vitro* findings.

We analyzed 230 patients with stage II/III sporadic colorectal cancers for which we had 5-FU treatment and survival data. Archival DNA was analyzed for EMAST (>2 of 5 markers mutated among UT5037, D8S321, D9S242, D20S82, D20S85 tetranucleotide loci). Kaplan-Meier survival curves were generated and multivariate analysis was used to determine contribution to risk.

We identified 102 (44%) EMAST cancers. Ninety-four patients (41%) received adjuvant 5-FU chemotherapy, and median follow-up for all patients was 51 months. Patients with EMAST CRCs demonstrated improved survival with adjuvant 5FU to the same extent as patients with non-EMAST CRCs (P<0.05). We observed no difference in survival between patients with stage II/III EMAST and non-EMAST cancers (P = 0.36).

There is improved survival for stage II/III CRC patients after adjuvant 5-FU-based chemotherapy regardless of EMAST status. The loss of contribution of hMSH3 for 5-FU cytotoxicity may not adversely affect patient outcome, contrasting patients whose tumors completely lack DNA MMR function (MSI-H).

## Introduction

Colorectal cancer (CRC) is one of the most common causes of cancer-related mortality worldwide [1]. In the United States alone, 50,000 people will die from CRC during 2014 [2]. Patients who lack distant CRC metastasis are potentially cured via surgery, and five-year survival rates for patients with stage II and stage III CRC approximate 75% and 55%, respectively [3, 4]. To minimize relapse of surgical therapy, adjuvant 5-fluorouracil (5-FU)-based chemotherapy has become standard for some high risk patients with stage II CRC and all patients with stage III CRC, as it improves survival rates by 10–20% [4, 5]. 5-FU exerts several metabolic actions that induce cytotoxicity in cancer cells, including (a) inhibition of thymidylate synthetase (which catalyzes the conversion of deoxyuracil monophosphate (dUMP) to deoxythymidine monophosphate (dTMP), leading to a deficiency of thymine nucleotides for DNA synthesis and subsequent substitution of uracil and 5-fluorouracil nucleotides for DNA synthesis, and (b) incorporation into RNA [5].

The incorporation of 5-FU into DNA is where the DNA mismatch repair (MMR) system recognizes, binds, and executes some of 5-FU’s cytotoxicity, independent of 5-FU’s effect on RNA [6–11]. The recognition complexes of DNA MMR, hMutSα and hMutSβ, consists of heterodimers of the MMR proteins hMSH2-hMSH6 and hMSH2-hMSH3, respectively [12, 13]. hMutSα recognizes single base mispairs after DNA synthesis and short insertion/deletion loops (I/D loops) at repetitive microsatellite DNA sequences, whereas hMutSβ recognizes larger I/D loops [12, 13]. Once a mispair or I/D loop is recognized, the execution complex hMutLα (a heterodimer of the MMR proteins hMLH1 and hPMS2) binds to hMutSα or hMutSβ to signal other proteins for excision and re-synthesis of the affected DNA, or commits the cell to programmed cell death if repair is futile [12, 13]. With respect to 5-FU, CRC cells completely deficient in DNA MMR are >20-fold more resistant to 5-FU cytotoxic killing [6]. Protein-DNA binding assays and surface plasmon resonance experiments demonstrate that hMutSα is the principal recognition complex for 5-FU incorporated in DNA as it has the highest affinity for binding and demonstrates the largest degree of cytotoxicity [8, 9]. The complex hMutSβ, despite its principle function in binding larger I/D loops, can also bind 5-FU and signal cytotoxicity, but to a lesser extent than hMutSα [9, 10]. When both hMutSα and hMutSβ are present, the highest level of 5-FU cytotoxicity is achieved, indicating that both DNA MMR recognition complexes synergize to contribute to 5-FU cytotoxicity, compared to hMutSα and hMutSβ alone [9, 10]. Thus, the DNA MMR recognition complexes have a hierarchical level for chemosensitivity for 5-FU cytotoxicity. Absence of both hMutSα and hMutSβ prevents the complexes to bind 5-FU within DNA, and lack of hMutLα (hMLH1-hPMS2 heterodimer) removes the execution component of MMR, with both scenarios causing complete absence of DNA MMR and aborting 5-FU-induced cytotoxicity [8–10].

These findings have implications for the ~15% of CRC patients with DNA MMR-deficient tumors who receive 5-FU therapy. We and others have previously shown that stage II/III sporadic CRC patients whose tumors demonstrate microsatellite instability-high (MSI-H) (i.e. are MMR-deficient) do not have improvement in their survival with the use of adjuvant 5-FU therapy, whereas patients whose tumors retain MMR improve their survival [14–19]. Thus, in CRC patients, tumor competency for DNA MMR is important for a survival response to 5-FU.

Elevated microsatellite alterations at selected tetranucleotide repeats (EMAST) is a genetic signature observed in up to 60% of CRCs [20–24]. The presence of EMAST is strongly associated with intraepithelial inflammation, advanced CRC stage, poor survival, and African American race [21, 22, 25]. EMAST CRCs show nuclear heterogeneous expression of hMSH3, [20, 22] and recent studies show that cytokine-driven oxidative stress induces a nuclear-to-cytosolic shift for hMSH3, removing it from its functional DNA repair site, and is the likely explanation for the observed nuclear heterogeneity [26, 27, 28]. It is theorized that inflammation and oxidative stress trigger hMSH3 mislocalization, causing hMutSβ function to fail, while still preserving hMutSα function in the nucleus [26]. EMAST and the mislocalization of hMSH3 is the most common DNA MMR defect in human CRC.

Our aim was to examine if survival of CRC patients was affected by 5-FU therapy if they possessed EMAST tumors. This was an important question as adjuvant 5-FU chemotherapy is the staple base therapy for patients with stage II/III CRC, and 60% of patients have acquired hMutSβ dysfunction due to hMSH3 mislocalization.

## Materials and Methods

### Patient samples and DNA extraction

We analyzed 230 patients with stage II/III sporadic colorectal cancers from University of California and Veterans Administration Medical Centers in San Diego, California (collected from 1982–1989) [14] and Hospital General Universitario de Alicante, Alicante, Spain (EPICOLON, collected from 2000–2001) [15, 16]. The University of California San Diego, VA San Diego, Hospital General Universitario de Alicante, and the University of Michigan Human Subjects Committees approved the research utilizing these existing pathological specimens. Patient records/information were anonymized and de-identified prior to analysis of data. Because of this use of existing pathological specimens that are de-identified, the institutional review boards waived the need for patient or next of kin consent. Patients were only included for this cohort if pathological material was available, their chemotherapeutic regimen known, and survival information was available. All patients underwent curative surgical resection. Patients were assigned to 5-FU based treatment or no treatment by their care teams prior to our study, and tumor MSI status was previously determined [14–16]. **Table A in S1 File** details all patient and tumor profiles in total. Tumor location, patient age, patient sex, tumor stage, use of chemotherapy and survival were derived from the surgical pathology report, with follow-up data retrieved from the tumor registries from the respective institutions [14–16]. Formalin-fixed, paraffin-embedded blocks were obtained for each patient, and serial sections cut for H&E staining and microdissection. DNA was isolated from each patient’s colorectal tumor and adjacent normal tissue. Each area microdissected was identified on a reference H&E staining slide. Dissected specimens was deparaffinized in a microfuge tube with xylene, and DNA was purified with ethanol and QIAmp DNA Investigator Kit (Qiagen, Valencia, CA) following a previous published protocol [22].

### DNA amplification, EMAST determination, and fragment analysis

EMAST status was determined using 5 polymorphic tetranucleotide markers (*UT5037*, *D9S242*, *D20S85*, *D8S321*, and *D20S82*) [22]. Genomic DNA extracted from tumors and counterpart normal tissues were PCR-amplified by specific primers for each tetranucleotide marker using Platinum PCR Supermix (Invitrogen, USA) as per the manufacturer’s protocol. PCR was performed using 6-FAM or HEX labeled primers. Cycling conditions were as follows: 95°C for 15 minutes for the initial heat activation; 40 cycles of 94°C for 1 minute, 55 C°–62C° for 1 minute, and 72°C for 30 seconds; and a final extension at 72°C for 10 minutes. Fluorescently labeled fragments generated by PCR were analyzed on an Applied Biosystems 3730xl DNA Analyzer with the GeneMarker (SoftGenetic LLC, PA). PCR products were used for DNA fragment analysis to identify frameshift mutation at tetranucleotide repeats for each locus. When aberrant peaks +/- multiples of 4 nucleotides were observed in the electrophoretograms from tumor as compared to that of the paired non-tumor, the marker was listed positive for frameshift instability. Tumors showing frameshifts in at least 2 tetranucleotide markers compared to paired normal tissue were categorized as EMAST-positive tumors, whereas all others were categorized as EMAST-negative tumors.

### Statistical analysis

Statistical analyses were conducted using SPSS (version 20, IBM) and by the Biostatistics Department, School of Public Health, University of Michigan. The following variables were assessed: age, sex, colonic location of tumor, stage, use of adjuvant 5-FU therapy, follow-up time, and vital status (alive or dead). Location of the tumor was classified as proximal (right) if the tumor was at or proximal to the splenic flexure, and distal (left-sided) if distal to the splenic flexure. Disease stage was classified at the time of surgery. Vital status was classified as overall survival. The statistical association between EMAST and other categorical variables was evaluated using Fisher’s exact test. Survival analyses were performed after patients were classified on the basis of their EMAST status and whether they received adjuvant 5-FU chemotherapy as part of their treatment. To examine differences in survival rates, Kaplan–Meier curves were plotted and the Cox proportional hazard function allowed calculation of the relative hazard risk ratio. All statistical tests were two-sided, and *P* values <0.05 were considered to be statistically significant.

## Results

### Patient characteristics and EMAST status

We utilized 230 stage II/III CRC patients from EPICOLON [15, 16] and USA [14] sources for which we had survival outcome, adjuvant 5-FU utilization, and tissue available for genetic analysis. For all 230 patients segregated by stage, 64 of 118 (54%) stage III patients received adjuvant 5-FU therapy, compared to 30 of 112 (27%) stage II patients (*P* = 0.000023). Of the 230 cancers analyzed for EMAST, 102 (44.3%) were EMAST-positive, defined as 2 or more tetranucleotide markers showing frameshift mutation [22]. We compared patients with EMAST cancers with those who had non-EMAST cancers, as shown in **Table A in S1 File**. We found no significant differences between EMAST and non-EMAST CRC patients for gender, intra-colon tumor location, or stage. However, patients with non-EMAST cancers were more often treated with 5-FU (*P*<0.05) (**Table A in S1 File**). This observation for patients with non-EMAST cancers might be predicted to favor enhanced survival compared to patients with EMAST cancers, as this group of patients possesses DNA MMR-proficient cancers. We found more MSI-H tumors among EMAST CRC patients (**Table A in S1 File**). This finding is likely due to complete inactivation of DNA MMR (hypermethylation of *hMLH1* inactivating the hMutLα complex in sporadic MSI-H tumors), which itself can cause frameshift mutations at tetranucleotide repeats, and/or secondary inactivation of *hMSH3* through frameshift mutation of its coding microsatellite as a consequence of the loss of hMutLα function [12].

### Overall survival of patients with EMAST and non-EMAST CRCs

We ascertained if CRC patients with EMAST (whose cancers are presumed missing hMutSβ function) would have a decrement in survival compared to CRC patients whose tumors retain MMR-proficiency (retaining both hMutSα and hMutSβ function, as well as hMutLα function) after adjuvant 5-FU therapy.

Of the 230 stage II/III CRC patients, 94 (40.9%) patients died over the median follow up time of 51.0 months. Because our true comparison was for survival of CRC patients with presumed loss of hMSH3 (hMutSβ) function compared to CRC patients who retain hMutSα and hMutSβ and hMutLα function (full DNA MMR proficiency), we analyzed our survival data in the absence of the 30 patients (13%) with MSI-H CRCs (all with hMutLα dysfunction). Removing patients with MSI-H CRCs also reduces confounding issues with outcome as it is well demonstrated that these patients do not respond favorably to 5-FU adjuvant therapy [14–19]. Additionally, comparing the 11 MSI-H CRC patients who received adjuvant 5-FU to the 19 MSI-H CRC patients who did not receive adjuvant 5-FU, there was no difference in survival (*P*>0.05, data not shown), consistent with prior multiple reports [14–19]. This left a total of 200 patients (82 EMAST and 118 non-EMAST) for analysis, and their characteristics are presented in **Table 1**. Without inclusion of the MSI-H CRC patients, the difference in 5-FU utilization between EMAST and non-EMAST patients disappeared.

**Table 1:** Clinical characteristics of CRC patients segregated by EMAST status, exclusive of MSI-H patients.

	All	EMAST	Non-EMAST	
	(*N* = 200)	(*N* = 82)	(*N* = 118)	*P* value
Age (years), median (range)	71 (22–93)	71 (40–93)	71 (22–93)	
Gender				
Female	85	29	56	
Male	115	53	62	0.0889
Tumor site				
Proximal	71	26	45	
Distal	129	56	73	0.350
5-FU chemotherapy				
With	83	28	55	
Without	117	54	63	0.0785
Stage				
II	91	38	53	
III	109	44	65	0.842

**Fig 1:**
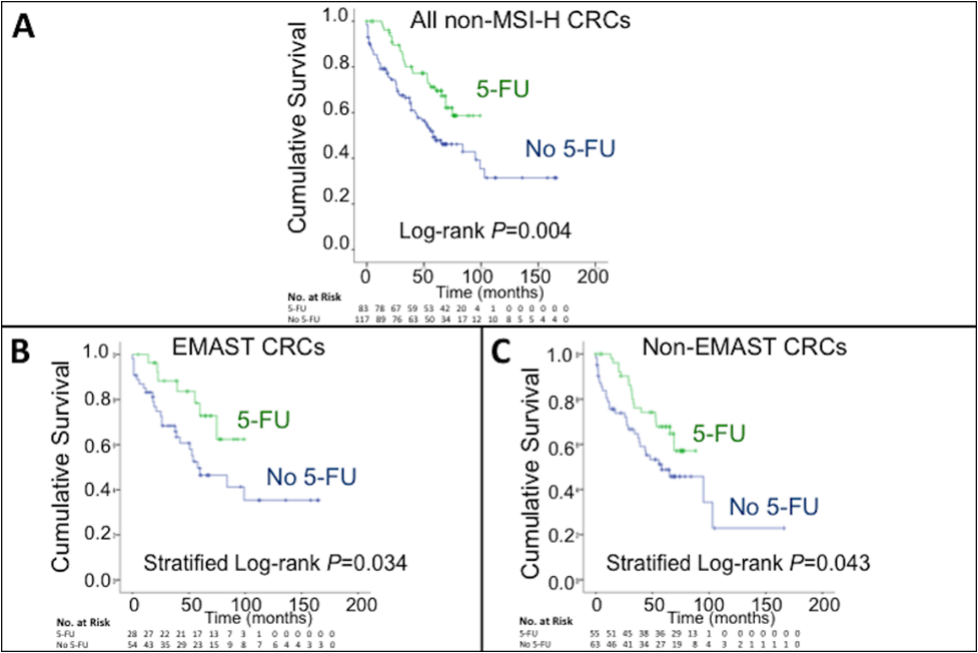
Kaplan-Meier plots of cumulative overall survival in patients treated with and without adjuvant 5-FU chemotherapy. (*A*) Cumulative overall survival of all patients without MSI-H (*P* = 0.004, log-rank test). (*B*) Cumulative overall survival of patients with non-MSI-H EMAST-positive tumors (deficient in hMSH3 function) (*P* = 0.034, stratified log-rank test). (*C*) Cumulative overall survival of patient with non-MSI-H, non-EMAST tumors (full competency in DNA mismatch repair function) (*P* = 0.043, stratified log-rank test).

We constructed Kaplan-Meier curves to compare cumulative overall survival in patients treated with and without adjuvant 5-FU chemotherapy. Adjuvant 5-FU adjuvant improves overall survival for patients with stage II/III CRC (*P* = 0.004, log-rank test) (**Fig 1A**), as has been previously reported [14,19]. We then segregated the data by the CRC EMAST status. As shown in **Fig 1B**, patients whose cancers demonstrated EMAST (presumed hMSH3-deficient) had improved survival with adjuvant 5-FU therapy (*P* = 0.034) to the same extent as patients whose cancers did not demonstrate EMAST (MMR-proficient) (*P* = 0.043) (**Fig 1C**). Thus, despite the lack of hMSH3 function within the cancer, there is improved survival in stage II/III CRC patients after adjuvant 5-FU chemotherapy regardless of tumor EMAST status.

### Risk factors affecting overall survival

Our cohort of CRC patients did not show worse survival if the patient had an EMAST cancer compared to a non-EMAST cancer, regardless of the presence or absence of adjuvant 5-FU treatment (**Fig. A in S1 File**). This contrast to prior reports of patients with EMAST cancers showing advanced stage and worse prognosis over patients with non-EMAST cancers [21, 25] could be due to the high number of stage II patients examined in our study. To validate this observation in our cohort of patients, we conducted a multivariate Cox proportion hazard analysis for overall survival (**Table 2**). From the analysis, we observed no survival difference between stage II/III CRC patients with EMAST and non-EMAST cancers in our cohort (*P* = 0.36). We also observed no difference for gender, intra-colonic tumor location, and the source of the patient for our cohort. We did observe significant worse survival for patients older than 70 years of age (*P* = 0.015), if the patient had a stage III tumor compared to a stage II tumor at the time of surgery (*P* = 0.0042), and if the patient did not receive adjuvant 5-FU chemotherapy (*P* = 0.0004).

**Table 2:** Multivariate *Cox* proportional Hazard Analysis of Risk Factors for overall survival.

Variable	Risk ratio	95% CI	*P* value
Age (≥71 vs. <70)	1.74	1.12 to 2.72	**0.015**
Gender (female vs. male)	1.19	0.77 to 1.84	0.43
Tumor site (proximal vs. distal)	1.09	0.70 to 1.70	0.71
Stage (III vs. II)	1.92	1.23 to 3.00	**0.0042**
5-FU treatment (5-FU vs. none)	0.40	0.25 to 0.67	**0.0004**
Cohort (EPICOLON vs. USA)	1.18	0.73 to 1.88	0.50
EMAST (EMAST vs. non- EMAST)	0.82	0.53 to 1.26	0.36

## Discussion

Our data for 230 Stage II/III CRCs segregated by EMAST status indicates that 5-FU remains beneficial for overall survival for patients with EMAST CRCs. The loss of hMutSβ function due to the mislocalization of hMSH3 [26] in EMAST CRCs appears not to be detrimental enough to modify patient outcome in response to adjuvant 5-FU therapy. Previously, we have shown a strong correlation between hMutSα and hMutSβ binding of 5-FU within DNA and 5-FU cytotoxicity, and when DNA MMR function was absent (either with loss of hMutSα and hMutSβ or hMutLα), there was no binding and no subsequent execution of cytotoxicity [8–10]. Multiple studies show adjuvant 5-FU treatment of patients with MSI-H CRCs translate the *in vitro* observation of lack of cytotoxicity into a lack of improved patient survival with 5-FU therapy [14–19]. The recognition of 5-FU in DNA appears to be predominantly by hMutSα, with affinity for binding of 5-FU more than twice that of hMutSβ in surface plasmon resonance assays [8, 9], paralleling hMutSα’s larger role in 5-FU-induced cell death [9, 10]. Although hMutSβ can bind 5-FU within DNA [9, 10] and its presence can induce a moderate level of cytotoxicity alone as well as synergism with hMutSα after 5-FU treatment [9], its non-functional status as determined by EMAST within patient CRCs that still possess hMutSα function appears not to be wholly critical to the success of 5-FU cytotoxicity on patient survival.

DNA MMR defective tumors can occur in the setting of sporadic CRCs (hypermethylation of *hMLH1*) as well as in Lynch syndrome, in which germline mutation of a DNA MMR gene is transmitted [12, 13, 29]. The MMR genes *hMLH1*, *hPMS2*, and *hMSH2* (and *EPCAM*, which secondarily targets inactivation of *hMSH2*) when mutated in the germline and the second allele somatically inactivated in the tumor completely inactivates DNA MMR function, generates an MSI-H cancer [30], and patients do not derive a benefit from adjuvant 5-FU therapy [17]. There has been no description of Lynch syndrome patients with a germline hMSH3 mutation to date to test the effect of adjuvant 5-FU treatment on outcome for this group, and perhaps their outcome would mirror patients with EMAST CRCs treated with 5-FU due to the remaining and functional hMutSα complex. However, we would predict that Lynch syndrome patients with *hMSH6* germline mutations would have a marked reduction in survival outcome with treatment for CRC compared to patients who retain MMR function in their tumors due to loss of hMutSα. Since *hMSH6* germline Lynch patients retain hMutSβ function, we would predict a muted response to adjuvant 5-FU that was better than patients whose tumors completely lack MMR function.

We did not observe a difference in overall survival between patients with EMAST and non-EMAST CRCs contrary to other reports [25], by stage and regardless of adjuvant 5-FU therapy. We believe this is largely due to the higher proportion of stage II patients in our study as compared to other studies, with nearly 50% of patient CRCs as stage II and who have a better survival outcome [3, 5]. The collection dates for our CRCs in our cohorts may have skewed the stage as well the number of persons receiving adjuvant 5-FU therapy. We only compared EMAST vs. non-EMAST CRC patients, and did not combine MSI-L CRC patients with EMAST CRC patients as other studies [25]. Indeed, the analysis of EMAST vs. non-EMAST CRC patients alone, without MSI-L CRC patients, show no survival differences [25]. Lastly, we examined overall survival compared to recurrence free survival [25]. Although EMAST been partly correlated with TNM stage [21], but has not specifically been correlated with venous, perineural, or lymphatic invasion, it is correlated and has a higher frequency with progression through the adenoma-to-carcinoma morphology progression, and in ulcerated carcinomas [22, 31]. We also performed our analysis of patients with EMAST CRCs without the influence of patients with MSI-H CRCs, all of whom have better survival compared to patients with non-MSI-H CRCs [12, 13] even without the use of 5-FU therapy.

A limitation of our observational study is that we were not able to determine recurrence-free or disease-specific survival due to lack of full information for this combined cohort. However, we are confident in our EMAST vs. non-EMAST comparison for overall survival as the stratified log-rank test confirms no difference in outcome controlling for 5-FU treatment status (**Table B in S1 File**), and an alternative length of survival analysis shows distribution similarities between EMAST and non-EMAST groups (**Fig. B in S1 File**).

In conclusion, stage II/III patients with EMAST CRCs respond just as well to adjuvant 5-FU chemotherapy with an improvement in their overall survival as do patients with non-EMAST tumors. This was an important question to examine that could have changed the approach to adjuvant chemotherapy for 60% of CRC patients. This is in contrast to patients with MSI-H CRCs, whose tumors have ineffectual hMutSα and hMutSβ or hMutLα complexes and who do not improve their survival with adjuvant 5-FU therapy [14–19]. The presence of intact hMutSα function (along with hMutLα function) in EMAST CRCs appears to be adequate to contribute towards an improved overall outcome for patients treated with adjuvant 5-FU.

## Supporting Information

S1 File Clinical characteristics of CRC patients segregated by EMAST status, inclusive of MSI-H patients (Table A). Stratified log-rank test for comparing EMAST and 5-FU status (**Table B**). Kaplan-Meier plots of cumulative overall survival in patients segregated by EMAST status (**Fig. A**). Alternative survival plots for EMAST and non-EMAST patients (**Fig. B**).
(PDF)
