# Supplementary material for: Efficacy of Adjuvant 5-Fluorouracil Therapy for Patients with EMAST-Positive Stage II/III Colorectal Cancer
Source: PLoS One. 2015 May 21;10(5):e0127591. doi: 10.1371/journal.pone.0127591 (PMC4440728; doi:10.1371/journal.pone.0127591)
Supplement: S1 File — Stratified log-rank test for comparing EMAST and 5-FU status (Table B). Kaplan-Meier plots of cumulative overall survival in patients segregated by EMAST status (Fig. A). Alternative survival plots for EMAST and non-EMAST patients (Fig. B). (PDF) [file pone.0127591.s001.pdf]

SUPPLEMENTAL FILE

Efficacy of Adjuvant 5-Fluorouracil Therapy for Patients with EMAST-Positive Stage II/III Colorectal Cancer

Yasushi Hamaya[1], Carla Guarinos[1,2], Stephanie S. Tseng-Rogenski[1], Moriya Iwaizumi[1], Ritabrata Das[3], Rodrigo Jover[2], Antoni Castells[4], Xavier Llor[5], Montserrat Andreu[6], John M. Carethers[1]

[1]*Division of Gastroenterology, Department of Internal Medicine, University of Michigan, Ann Arbor, Michigan, United States of America;* [2]*Unidad de Gastroenterologia, Hospital General Universitario de Alicante, Alicante Spain;* [3]*Biostatistics Department, School of Public Health, University of Michigan, Ann Arbor, Michigan, United States of America;* [4]*Department of Gastroenterology, Hospital Clínic, Centro de Investigación Biomédica en Red en Enfermedades Hepáticas y Digestivas (CIBERehd), IDIBAPS, University of Barcelona, Barcelona, Spain;* [5]*Division of Gastroenterology, Department of Medicine, Yale University, New Haven, Connecticut, United States of America;* [6]*Department of Gastroenterology, Hospital del Mar, IMIM (Institut Hospital del Mar d'Investigacions Mèdiques), Pompeu Fabra University, Barcelona, Catalonia, Spain*

Supplemental Tables

Table A. Clinical characteristics of CRC patients segregated by EMAST status, inclusive of MSI-H patients.

	All (*N*=230)	EMAST (*N*=102)	Non-EMAST (*N*=128)	*P* value
Age (years), median (range)	71 (22-93)	71 (40-93)	71 (22-93)	
Sex				
Female	96	37	59	
Male	134	65	69	0.141
Tumor site				
proximal	95	42	53	
distal	135	60	75	1.00
5-FU chemotherapy				
With	94	34	60	
Without	136	68	68	**0.0433**
Stage				
II	112	51	61	
III	118	51	67	0.791
MSI status				
MSI-H	30	20	10	
MSI-L/MSS	200	82	118	**0.00832**

MSI=microsatellite instability; H=high; L=low, MSS=microsatellite stable

Table B. Stratified log-rank test for comparing EMAST and 5-FU status.

All CRCs—*P*-values (n=230)

	5-FU + no EMAST	no 5-FU + EMAST
5-FU + EMAST	0.471	0.0186
no 5-FU + no EMAST	0.0196	0.58

CRCs without MSI-H—*P*-values (n=200)

	5-FU + no EMAST	no 5-FU + EMAST
5-FU + EMAST	0.474	0.034
no 5-FU + no EMAST	0.043	0.68

Supplemental Figures

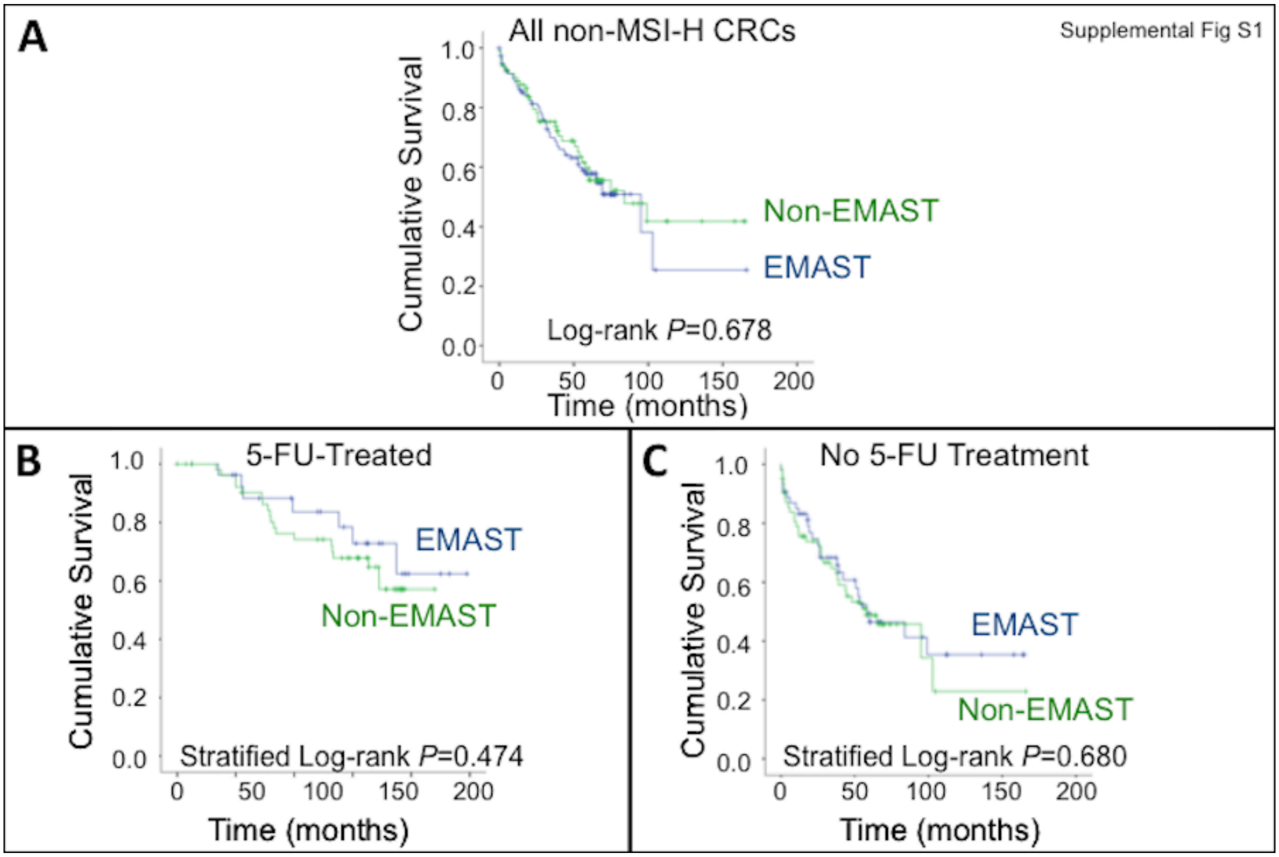

Fig. A. Kaplan-Meier plots of cumulative overall survival in patients segregated by EMAST status. (*A*) Cumulative overall survival of all patients without MSI-H separated by EMAST status (P=0.678, log-rank test). (*B*) Cumulative overall survival of all non-MSI-H patients receiving adjuvant 5-FU separated by EMAST status (P=0.474, stratified log-rank test). (*C*) Cumulative overall survival of all non-MSI patients who did not receive adjuvant 5-FU chemotherapy, separated by EMAST status (P=0.680, stratified log-rank test).

Supplemental Fig S2

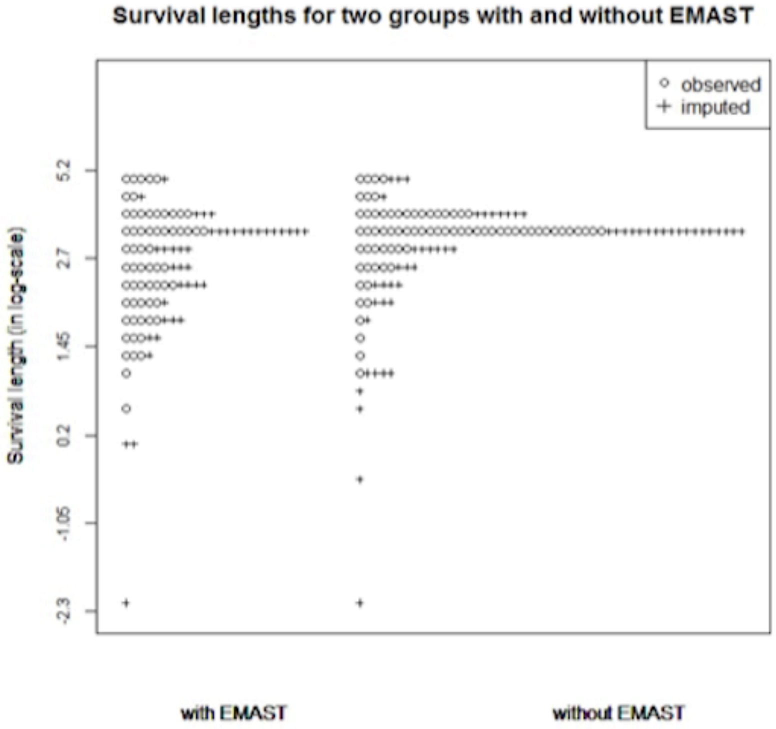

Fig. B. Alternative survival plots for EMAST and non-EMAST patients, according to Royston *et al*. *J Natl Cancer Inst*. 2008;100:92-97.
